# Hunting for Complex Organic Molecules in the Interstellar Medium: The Role of Accurate Low-Cost Theoretical Geometries and Rotational Constants

**DOI:** 10.1021/acs.jpca.3c06649

**Published:** 2023-11-30

**Authors:** Vincenzo Barone, Federico Lazzari

**Affiliations:** Scuola Normale Superiore, di Pisa, Piazza dei Cavalieri 7, Pisa 56125, Italy

## Abstract

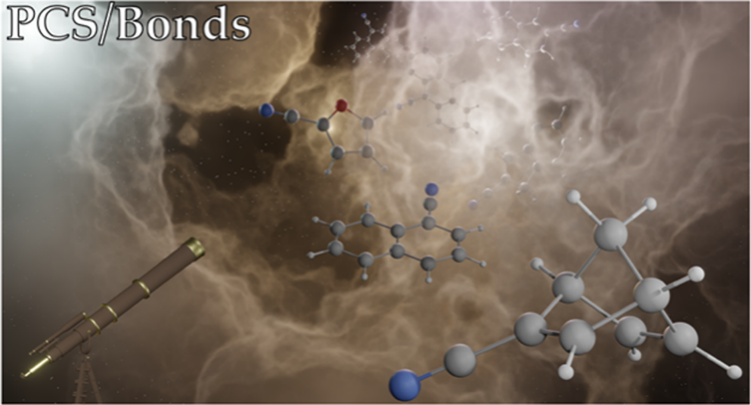

A new approach to computation at affordable cost of accurate geometrical structures and rotational constants for medium-sized molecules in the gas phase is further improved and applied to a large panel of interstellar complex organic molecules. The most distinctive feature of the new model is the effective inclusion of core–valence correlation and vibrational averaging effects in the framework of density functional theory (DFT). In particular, a double-hybrid functional in conjunction with a quadruple-ζ valence/triple-ζ polarization basis set is employed for geometry optimizations, whereas a cheaper hybrid functional in conjunction with a split-valence basis set is used for the evaluation of vibrational corrections. A thorough benchmark based on a wide range of prototypical systems shows that the new scheme approaches the accuracy of state-of-the-art wave function methods with the computational cost of the standard methods (DFT or MP2) routinely employed in the interpretation of microwave spectra. Since the whole computational workflow involves the postprocessing of the output of standard electronic structure codes by a new freely available web utility, the way is paved for the accurate yet not prohibitively expensive study of medium- to large-sized molecules also by nonspecialists.

## Introduction

The characterization of molecular structures and properties in the gas phase plays a central role in different fields ranging from molecular astrophysics and astrochemistry to the impact of atmospheric chemistry on climate change, not to mention combustion processes. From another point of view, the disentanglement of intrinsic stereoelectronic and environmental effects in processes occurring in condensed phases requires preliminary knowledge of gas-phase results. In this framework, the pivotal role of rotational spectroscopy is related to its highly selective nature: the observed spectral patterns depend sensitively on three rotational constants that, in turn, are inversely related to the principal moments of inertia of the target molecule. Furthermore, the resolution of spectrometers, either in a laboratory or through a telescope, is on the order of parts per million, meaning that the assignment of a molecule based on the rotational spectrum is usually unambiguous. Thanks to the development of spectrometers coupling supersonic-jet expansion^1^ and laser ablation,^2^ rotational spectroscopy can be applied to molecules with up to about 50 atoms including thermolabile molecules with high melting points (like several biomolecule building blocks). However, as the dimensions of the molecule increase, the spectral congestion grows due to both intrinsic factors and the presence of different low-energy species separated by sufficiently high-energy barriers to avoid their interconversion under the experimental conditions.^3,4^ Quantum chemical (QC) computations play a fundamental role in the disentanglement of these complexities, but the accuracy requirements of rotational spectroscopy are very stringent. As a consequence, state-of-the-art methods are needed, which are, unfortunately, applicable to systems containing at most a dozen atoms.^5−7^ For larger systems, the current standard is to use B3LYP or MP2 computations with medium-size basis sets.^8−11^ However, the intrinsic errors of these approaches (0.5–1%) are too large to allow unbiased analyses without additional experimental pieces of information (e.g., quadrupole coupling constants in the presence of nuclei, in which they do not vanish).^12^ Improved results can be obtained correcting the density functional theory (DFT) geometrical parameters by bond-specific parameters^13^ derived from a large database of accurate molecular structures^14,15^ [the so-called linear regression (LR) approach], or with reference to suitable fragments whose accurate geometries are already known [the so-called template molecule (TM) approach].^16^ Recently, the LR and TM approaches have been combined with remarkable success in the nano-LEGO model^14,15^ (also called LEGO-brick^17,18^). However, the use of a large number of parameters remains quite unsatisfactory and, above all, suitable fragments are not always available.^19,20^ Based on these premises, it is the purpose of this paper to show that much better results can be obtained by the recent Pisa composite scheme (PCS),^19,21−23^ which improves the accuracy of current approaches for medium-sized molecules by nearly an order of magnitude, without any significant increase of computational cost, especially when core–valence (CV) correlation is accounted for by a simple one-parameter approach (PCS/Bonds). We will consider in particular polyaromatic hydrocarbons (PAHs),^24^ together with some heteroaromatic derivatives (PANHs)^25^ and CN-substituted species.^26^ The last class of compounds is especially significant in astrochemistry since the presence of the CN moiety strongly increases the vanishing (or nearly vanishing) dipole moment of PAHs, thus allowing their characterization by means of rotational spectroscopy.^27,28^

## Methods

### PCS/Bonds Model

The key feature of the reduced cost composite methods developed in our group during the past few years^29−32^ is that starting from an accurate evaluation of valence energy (*E*_V_) with a (partially augmented) triple-ζ basis set,^33,34^ complete basis set (CBS) extrapolation and inclusion of CV correlation can be performed separately with good accuracy and negligible additional cost by means of second order Møller–Plesset perturbation theory (MP2).^35^ An equivalent partitioning of the different contributions starts, instead, from the valence energy computed at the MP2 level (*E*_V2_) and then adds post-MP2 valence contributions (Δ*E*_V_) and MP2 CV correlation (Δ*E*_CV2_)
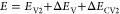
1with
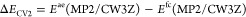
2where ae means all-electrons, fc means frozen-core, and CW3Z is the correlation-consistent cc-pwCVTZ basis set.^36^ This formulation permits the use of several computational models to estimate the Δ*E*_V_ contribution and the use of different basis sets (possibly also CBS extrapolations) for evaluating both *E*_V2_ and Δ*E*_V_. Derivation of eq 1 wrt Cartesian coordinates leads to energy gradients that can be employed for geometry optimizations by composite methods.^37,38^ However, a much simpler approach (referred to as the geometry scheme^7,39^) is obtained assuming that the additivity approximation can be directly applied to geometrical parameters (*r*) and only requires geometry optimizations at different levels of theory
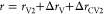
3

Several benchmarks have shown that gradient and geometry schemes provide nearly identical results,^40^ possibly due to the smallness (a few mÅ) of the corrections to the starting geometrical parameters. Furthermore, the geometry scheme does not require any modification of standard electronic structure codes, is embarrassingly parallel, and is particularly effective since low-scaling methods or analytical implementations are widely available for MP2 (or double-hybrid density functionals) gradients.

For systems not involving strong dynamical correlation, the coupled-cluster ansatz including single, double, and (perturbatively) triple excitations (CCSD(T))^41^ can be confidently employed to evaluate Δ*E*_V_ and Δ*r*_V_.^31,32^ However, remarkable results are obtained at a much reduced cost by evaluating the Δ*r*_V_ contribution with the rev-DSD-PBEP86-D3(BJ) double-hybrid functional^42^ (hereafter rDSD) in conjunction with the same basis sets employed for *r*_V2_.^21^ The final PCS/DFT model is obtained neglecting the CBS extrapolation and employing the cc-pVTZ-F12 basis set^43^ (hereafter 3F12), which involves a quadruple-ζ expansion of s, p valence orbitals in conjunction with a triple-ζ expansion of polarization functions. Under such circumstances, Δ*r*_V_ = *r*_rDSD_ – *r*_V2_ and eq 3 becomes
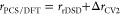
4

A further reduction of computational cost with negligible loss of accuracy is obtained by removing d functions on first-row atoms and replacing the two f functions on second- and third-row atoms by a single f function taken from the cc-pVTZ basis set.^33^ The resulting basis set (referred to as 3F12^–^ in the following) has dimensions comparable with those of the jun-cc-pVTZ basis set employed systematically in previous studies,^31^ but it delivers results much closer to those of augmented quadruple-ζ basis sets^21^ which are, in turn, practically at the CBS limit.^44^ From another point of view, extensive benchmarks confirmed that CV corrections are entirely negligible for valence and dihedral angles,^22^ which are predicted accurately at the rDSD/3F12^–^ level. Improved bond lengths can then be obtained by the following recipe
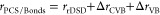
5where the first corrective term accounts for CV correlation and the second one stands for possible small inaccuracies in the treatment of valence electrons. Bonded atoms can be easily identified employing covalent radii (*r*^cov^, taken for instance from ref (45)) or, equivalently, Pauling bond orders *P*_*ij*_(46)
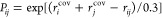
6In eq 6, *r*_*ij*_ is the interatomic distance in Å and two atoms are considered bonded if *P*_*ij*_ is larger than 0.3 (which corresponds to a distance 0.35 Å longer than the sum of the covalent radii). In a previous work,^22^ we have shown that Δ*r*_CVB_ is a simple function of the product (*n*_*ij*_) of the principal quantum numbers of the involved atoms. Here, a further step is taken by employing the same covalent radii already used in eq 6 in place of the rDSD/3F12^–^ bond lengths in order to obtain a method-independent estimate of the CV contributions (Δ*r*_CVB_)
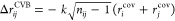
7

In the above equation, *k* = 0.0011 is the same as in ref (22), and vanishing corrections are correctly obtained for bonds between first-row atoms. In the case of the rDSD/3F12^–^ model, the Δ*r*_VB_ correction is needed only for multiple CC bonds, with the following simple expression having the correct behavior and remarkable numerical performance
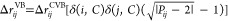
8

The Kronecker δ permits the inclusion of the corrective term only for specific bonds, and the precise values of the bond orders are not critical, provided that they are close to 1, 2, and 3 for simple, double, and triple bonds. In the following sections, we show that the PCS/Bonds model can deliver accurate results by a single geometry optimization at the rDSD/3F12^–^ level.

While *Z*-matrix nonredundant internal coordinates are usually employed to define (and correct) bond lengths and valence angles,^18^ this approach is ill adapted to deal with intrinsic redundancies (e.g., cycles). As a consequence, a full set of redundant internal coordinates is preferable, which can be automatically derived when the bond pattern of the molecular system is defined.^47^ However, the displacements computed by eq 5 are finite, so that it is, in principle, impossible to modify all of the bond lengths while retaining the original values of the other internal coordinates. Furthermore, the transformation between (redundant) internal and Cartesian coordinates is nonlinear, making the coordinate conversion an iterative procedure. Contrary to the customary Newton procedure, which requires the generalized inverse of the Jacobian matrix B,^47^ we resorted to a penalty function approach in which the following function is minimized by a gradient-descent algorithm
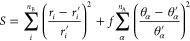
9where the index *i* labels the *n*_B_ bonds (with bond length *r*_*i*_), the index α labels the *n*_A_ angles θ, and *f* is a scale factor that normalizes the weight of bond lengths and angles (a default value of 0.00115 is used).

In particular, the Cartesian coordinates at the *i* + 1st iteration are
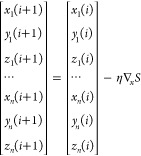
10where ∇_*x*_*S* is the Cartesian gradient of *S* and η is the so-called learning-rate in the machine learning (ML) jargon, with a value of around 0.1 performing well in the present context. The components of the gradient are
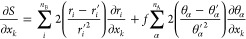
11

The derivatives of internal coordinates wrt Cartesian coordinates appearing in the previous equations are well-known^48^ and full convergence of the procedure (root-mean-square changes of Cartesian coordinates lower than 10^–6^) is obtained without problems in all the studied cases.

The whole procedure has been implemented in a Website, which, starting from a rDSD/3F12^–^ geometry in Cartesian coordinates (*xyz*- or Gaussian log-file), interactively produces the PCS/Bonds structure, the corresponding equilibrium rotational constants, and a 2D representation of the molecular structure.

## Results and Discussion

### Validation

In general terms, predicted rotational constants with an accuracy of 1% are not at all helpful for the analysis of samples containing several different species, whereas an accuracy of 0.01% would be extraordinarily valuable. The relationship between the accuracy of the rotational constants and that of the underlying geometrical parameters is not straightforward for polyatomic molecules. However, an accuracy between 0.0005 and 0.001 Å for bond lengths (in the range from 1 to 2 Å) and of about 0.1° for valence angles can be considered a reasonable guess for an accuracy of 0.1% on rotational constants.^49^ While this target is reachable by state-of-the-art composite wave function methods,^7,50,51^ the 0.01% goal would require consideration of additional effects (e.g., diagonal Born–Oppenheimer approximation and finite nuclear models) and also places essentially insurmountable demands on the basis set selection and correlation treatments.^49^ Based on these premises, we will analyze the perspectives of a low-cost model able to approach the 0.1% accuracy target for large molecules.

From another point of view, the current practice of comparing experimental rotational constants with computed equilibrium values neglects the contribution of the interaction between vibrations and rotations. For semirigid molecules, this contribution is well approximated employing second-order vibrational perturbation theory (VPT2)^52,53^
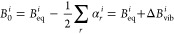
12where α_*r*_^*i*^ denotes the vibrational–rotation interaction constants, with *i* being the inertial axis (*a*, *b*, or *c*) and the sum running over all the *r* vibrational modes.

The magnitude of the vibrational contribution Δ*B*_vib_ ranges typically between 0.1 and 1.0% of the corresponding rotational constant (i.e., above the target accuracy of our model), and its calculation by QC methods requires evaluation of the harmonic and (semidiagonal cubic) anharmonic force fields. The error in such computations is comparable to that of the force constants, which is of the order of 10% for hybrid density functionals with medium-size basis sets like the B3LYP-D3(BJ)/6-31+G* model employed in the present context. Therefore, the average error in the vibrational contribution to the rotational constants can be estimated to be about 0.05% of the total value of the ground vibrational state constants *B*_0_, which is within the accuracy target (0.1%) discussed above.

In principle, the small electronic correction to rotational constants should also be considered^52^
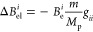
13where *g*_*ii*_ is expressed in units of the nuclear magneton, *m* is the electron mass, and *M*_p_ is the proton mass. Several studies have shown that Δ*B*_el_ can be safely computed by hybrid density functionals using London orbitals.^16,22^

Additional parameters of particular relevance for rotational spectra are the nuclear quadrupole coupling constants, together with quartic and sextic distortion constants, with the latter two quantities depending on harmonic and anharmonic force fields, respectively.^53^ Since the accuracy of the rDSD functional has been recently validated for all these quantities^54−57^ and also for vibrational transitions,^58,59^ we will not analyze again these aspects. The same remark applies to the components of dipole moments, which determine the intensities of rotational transitions.^60^

**Figure 1:**
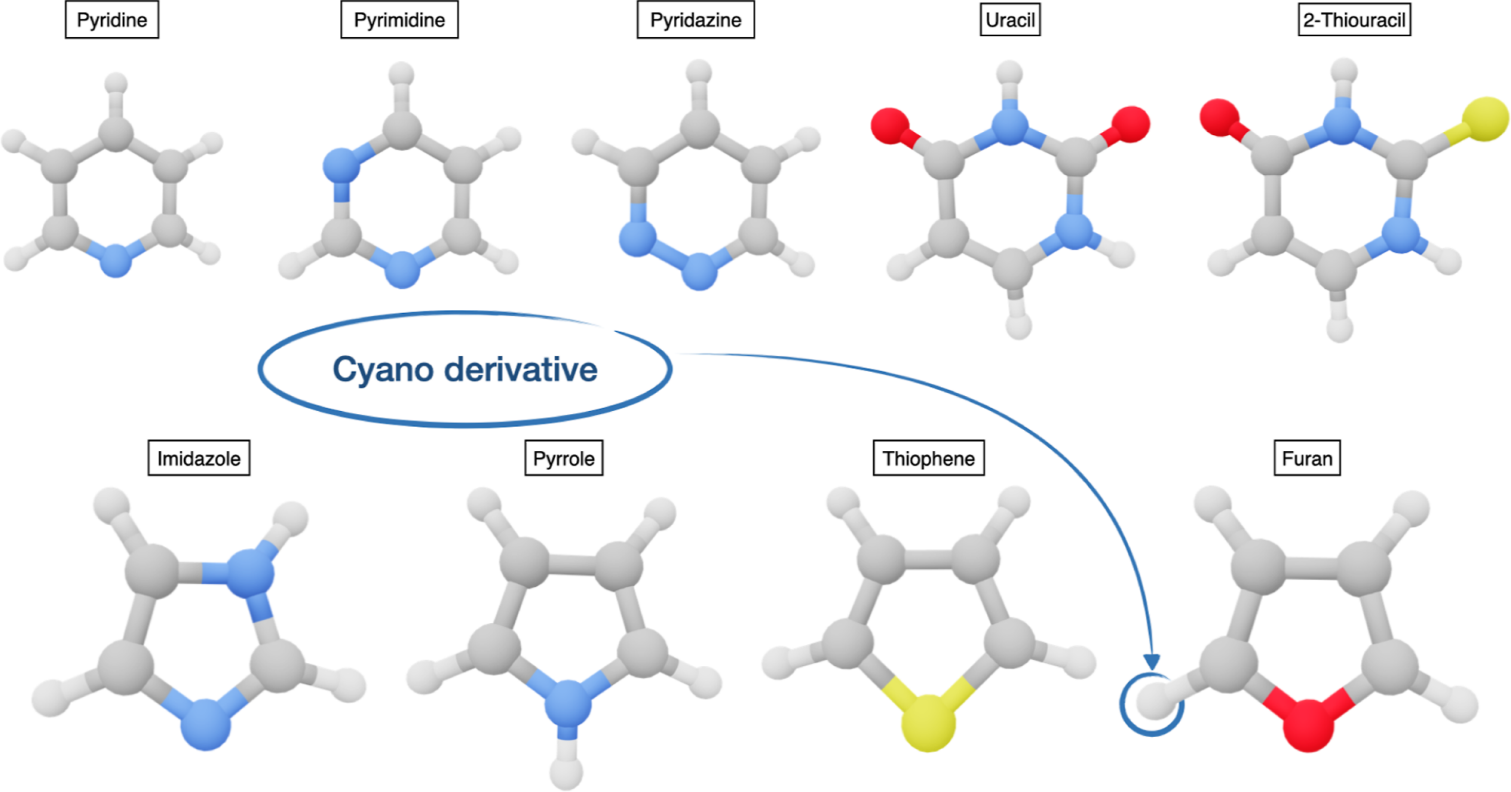
Structures of the molecules belonging to the validation set.

Before considering cyclic and bicyclic compounds of astrochemical interest, we analyze the performance of the new PCS/bonds model for a panel of medium-sized compounds (Figure 1) with the aim of determining the role of the different approximations and the weight of the various contributions.

The results collected in Table 1 show that the accuracy of the rDSD/3F12^–^ model is reasonable, but the experimental ground-state rotational constants are systematically overestimated. However, the straightforward interpretation of this result in terms of underestimated bond lengths (related to the inverse proportionality between rotational constants and inertia moments) is misleading because it completely neglects the contribution of vibrational corrections. As a matter of fact, Δ*B*_vib_ is always the largest corrective contribution, it is only partially counterbalanced by the positive contribution of the CV correlation, and it actually leads to a non-negligible reduction of the equilibrium value. The final PCS/Bonds results always fulfill the target accuracy of 0.1% without any underlying error compensation. In fact, the rigorous Δ*B*_CV2_ correction and its one-parameter Δ*B*_CVB_ approximation are always very close, while the additional Δ*B*_VB_ contribution vanishes in the absence of multiple CC bonds and corrects for the slight overestimation of conjugative effects typical of DFT methods. Moreover, the use of a double-hybrid functional in conjunction with a sufficiently large basis set permits one to avoid any bond-specific correction and, even more importantly, any correction to valence and dihedral angles.

**Table 1:** Computed and Experimental Rotational Constants (in MHz) of the Prototypical Compounds

molecule	param.	*B*_eq_ (rDSD)	Δ*B*_CVB_^a^	Δ*B*_VB_	Δ*B*_Vib_^b^	Δ*B*_el_	tot.^c^	exp.
pyridine	B_*a*_	6067.4	25.3	–5.1	–44.7	–0.3	6042.9	6039.3
	B_*b*_	5832.9	23.0	–5.1	–37.1	–0.3	5813.7	5804.9
	B_*c*_	2973.6	12.4	–2.6	–21.3	0.1	2962.1	2959.2
pyrimidine	B_*a*_	6304.9	25.1	–1.2	–43.8	–0.3	6285.0	6276.8
	B_*b*_	6098.2	24.0	–2.9	–40.2	–0.4	6079.1	6067.2
	B_*c*_	3099.9	12.2	–1.0	–21.8	0.1	3089.3	3084.4
pyridazine	B_*a*_	6278.9	24.0	–3.3	–48.2	–0.4	6251.4	6243.0
	B_*b*_	5988.6	23.7	–2.7	–37.5	–0.5	5972.1	5961.1
	B_*c*_	3065.2	11.9	–1.5	–22.2	0.1	3053.4	3048.7
pyrrole	B_*a*_	9174.2	34.9	–2.9	–73.7	–0.5	9132.5	9130.6
	B_*b*_	9042.5	33.8	–7.0	–69.8	–0.3	8999.5	9001.3
	B_*c*_	4554.0	17.1	–2.5	–36.7	0.2	4531.9	4532.1
imidazole	B_*a*_	9769.3	38.2	–0.2	–80.0	–0.5	9727.3	9725.3
	B_*b*_	9418.1	36.8	–4.4	–77.1	–0.6	9373.5	9374.0
	B_*c*_	4795.2	18.8	–1.2	–40.9	0.2	4771.9	4771.9
furan	B_*a*_	9493.0	35.6	–3.0	–78.7	–0.5	9446.9	9447.1
	B_*b*_	9287.1	34.7	–8.9	–65.4	–0.5	9247.5	9246.7
	B_*c*_	4694.5	17.5	–3.0	–38.3	0.1	4670.7	4670.8
2-furonitrile	B_*a*_	9259.8	34.7	–7.7	–67.6	–0.5	9219.2	9220.3
	B_*b*_	2028.5	8.2	–0.9	–7.0	–0.4	2028.8	2029.2
	B_*c*_	1664.0	6.7	–0.9	–7.4	0.1	1662.4	1662.0
thiophene	B_*a*_	8077.0	34.1	–3.2	–59.5	0.4	8048.4	8041.8
	B_*b*_	5434.1	24.6	–3.1	–29.6	0.2	5426.0	5418.1
	B_*c*_	3248.5	14.3	–1.6	–21.7	–0.1	3239.5	3235.8
uracil	B_*a*_	3899.7	15.3(15.2)	–1.3	–27.7	–0.1	3886.1	3883.9
	B_*b*_	2025.8	8.3(6.6)	–0.1	–10.6	–0.1	2023.4	2023.7
	B_*c*_	1333.2	5.5(4.7)	–0.2	–7.4	0.0	1331.0	1330.9
2-thiouracil	B_*a*_	3568.3	15.4(14.8)	–2.7	–22.9	–0.1	3558.1	3555.1
	B_*b*_	1316.3	6.4(5.8)	–0.4	–6.4	0.0	1315.9	1315.0
	B_*c*_	961.6	4.5(4.1)	–0.4	–4.9	0.0	960.8	960.0
MUE %		0.392					0.069	
MAX %		0.575					0.196	

a In parenthesis is the Δ*B*_CV2_ contribution.

b At the B3LYP-D3(BJ)/6-31+G* level.

c Not including Δ*B*_el_.

It is apparent that the Δ*B*_el_ contribution is smaller than the target accuracy of our computational approach, at least for the molecules considered in the present study (see Table 1). As a consequence, in the following, this contribution will be neglected.

An intuitive picture of the performance of a given model is offered by a graphical representation of the results based on normal distributions defined by
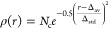
14

**Figure 2:**
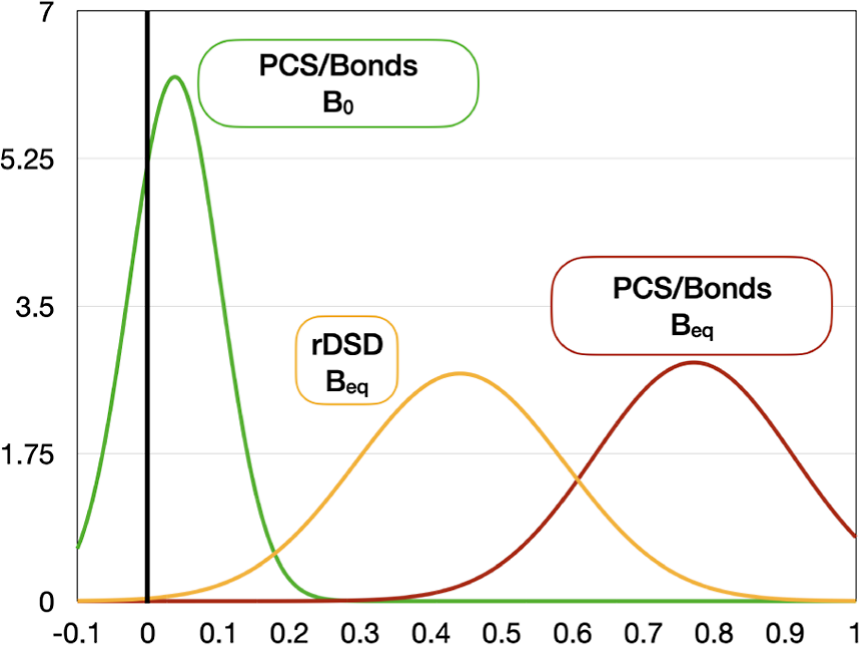
Error statistics for the semirigid molecules of the validation set.

In eq 14, Δ_av_ and Δ_std_ are the relative unsigned mean error and standard deviation, respectively, whereas *N*_c_ is a suitable normalization constant. The results shown in Figure 2 confirm the remarkable accuracy and robustness of the PCS/Bonds model.

**Figure 3:**
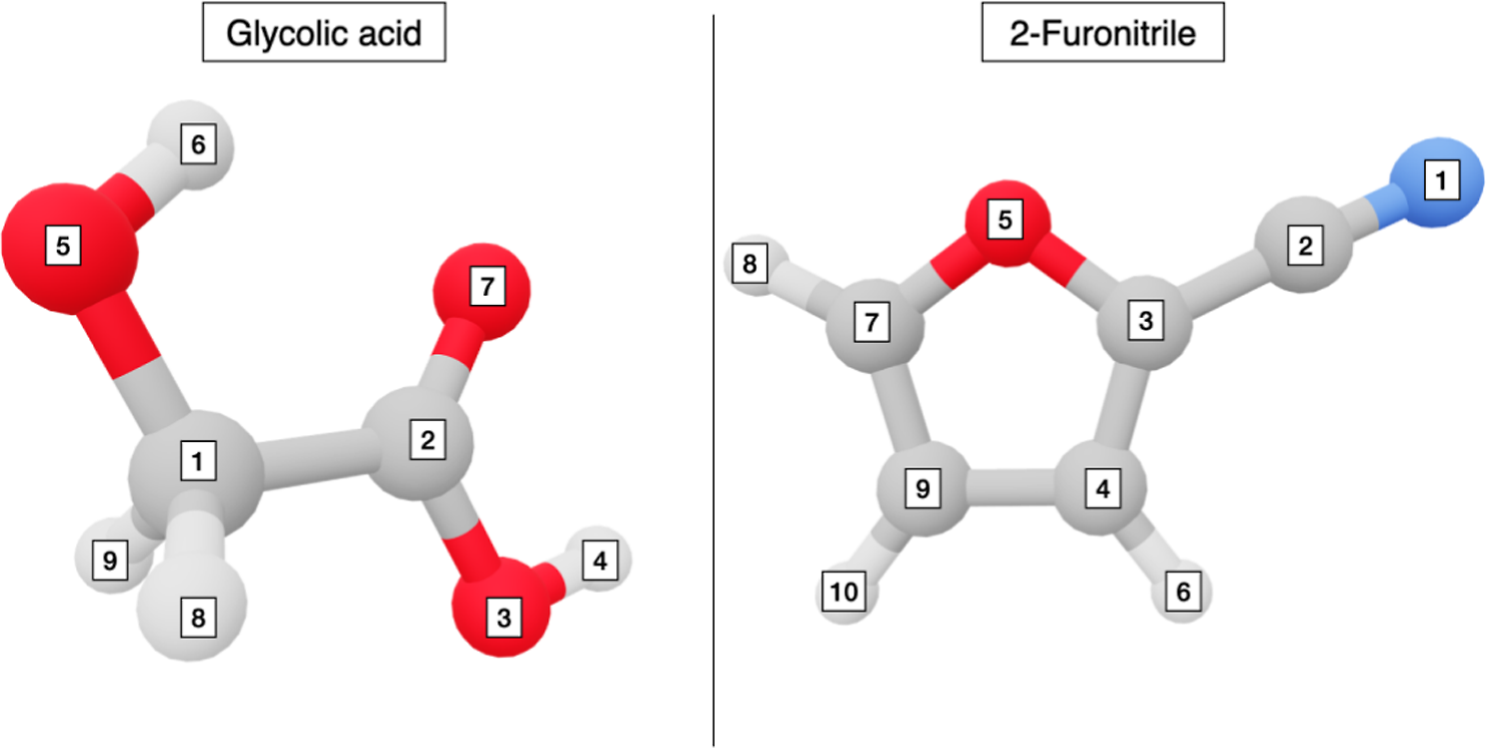
Structure and atom numbering of glycolic acid (most stable conformer) and 2-furonitrile.

While all the computed rotational constants are in remarkable agreement with experiment, it would be useful to have a direct comparison between experimental and computed geometrical parameters since, especially for medium-sized molecules, the final experimental outcome could be tuned by different factors. Unfortunately, accurate experimental structures are not available for the quite large PAHs, which are the main targets of the present study. However, the very recent determination of the semi-experimental equilibrium structure of 2-furonitrile^61^ (see Figure 3) permits a detailed comparison between PCS/Bonds and experimental geometrical parameters for a heteroaromatic molecule containing the CN moiety. The results collected in Table 2 show that the accuracy of all the geometrical parameters is in the expected range and rivals that of the composite “Cheap” wave function method,^31,62^ which requires orders-of-magnitude larger computer resources and, above all, is not applicable to large PAHs.

**Table 2:** Computed and Experimental Rotational Constants (in MHz) of 2-Furonitrile

param.	exp.^a^^,^^b^	Cheap^b^^,^^c^	rDSD^d^	PCS/Bonds^d^
C2–N1	1.1587	1.1574	1.1623	1.1595
C2–C3	1.4173	1.4177	1.4179	1.4157
C3–C4	1.3623	1.3627	1.3649	1.3633
C3–O5	1.3590	1.3597	1.3633	1.3606
O5–C7	1.3544	1.3535	1.3568	1.3541
C7–C9	1.3572	1.3566	1.3607	1.3591
C4–H6	1.0747	1.0747	1.0776	1.0765
C7–H8	1.0737	1.0737	1.0766	1.0754
C9–H10	1.0744	1.0744	1.0774	1.0762
<(C3C2N1)	179.70	178.59	178.65	178.65
<(C4C3C2)	131.06	131.59	131.75	131.75
<(C3C4O5)	110.93	110.90	110.94	110.94
<(C3O5C7)	106.24	106.22	106.36	106.36
<(O5C7C9)	111.07	110.90	110.94	110.94
<(C3C4H6)	125.93	125.93	125.91	125.91
<(O5C7H8)	115.65	115.65	115.65	115.65
<(C4C9H10)	127.47	127.47	127.43	127.43
<(C7C9H10)	126.49	126.49	126.56	126.56
B_*a*_^0^	9220.3	9235.1(0.16)	9192.2(0.30)	9219.2(0.01)
B_*b*_^0^	2029.3	2030.9(0.08)	2021.5(0.38)	2028.8(0.02)
B_*c*_^0^	1662.6	1664.2(0.10)	1656.6(0.36)	1662.4(0.01)

a From ref (61) with rotational constants truncated to the first decimal place.

b CH bond lengths, together with H6C4C3, H8C7O5, H10C9C4, and H10C9C7 valence angles fixed at the Cheap computed values.

c For the definition and expected accuracy of the Cheap model, see refs (17) and (62).

d This work.

While the main targets of the present study are cyclic and bicyclic molecules, the search for other similar-sized linear and complex molecules in space is also important, especially in exoplanet studies. In principle, the errors of the PCS/Bonds model should be the same for linear and cyclic molecules, but the former class of molecules is usually more flexible than the latter one. This feature increases the difficulty of accurate structure evaluations due to the presence of large amplitude motions, which limit the precise location of energy minima and the accuracy of the perturbative evaluation of vibrational corrections.^57^ In order to address these limitations for a typical flexible organic molecule, we report in Table 3 the geometrical parameters and spectroscopic constants for the most stable conformer of glycolic acid (see Figure 3).

**Table 3:** Computed and Experimental Rotational Constants (in MHz) of Glycolic Acid

param.	exp^a^	CCSD(T)^b^^,^^c^	rDSD^c^^,^^d^	PCS/Bonds^c^
C1–C2	1.505	1.511	1.509	1.506
C2–O3	1.339	1.345	1.342	1.340
O3–H4	0.966	0.968	0.967	0.966
C1–O5	1.399	1.404	1.402	1.399
O5–H6	0.965	0.967	0.966	0.965
C2–O7	1.205	1.210	1.207	1.204
C1–H9	1.090	1.095	1.095	1.093
<(C1C2O3)	112.6	112.6	112.4	112.4
<(C2O3H4)	106.9	106.4	107.1	107.1
<(C2C1O5)	110.8	110.7	111.1	111.1
<(C1O5H6)	106.5	105.4	106.7	106.7
<(C1C2O7)	124.0	124.0	123.6	123.6
<(C2C1H9)	108.0	108.0	108.2	108.2
B_*a*_^0^	10696.1	10600.7(0.84)	10626.8(0.64)	10670.2(0.24)
B_*b*_^0^	4051.0	4025.9(0.58)	4024.1(0.66)	4038.8(0.30)
B_*c*_^0^	2994.7	2974.5(0.65)	2975.0(0.65)	2986.2(0.28)

a Experimental ground-state rotational constants from ref (56) truncated to one decimal place.

b In conjunction with the cc-pVTZ basis set.

c The vibrational corrections (taken from ref (56)) are 96.9, 45.8, and 30.4 MHz.

d In conjunction with the 3F12^−^ basis set.

Comparison with the accurate semi-experimental equilibrium structure^56^ shows that the average error of PCS/Bonds bond lengths and valence angles are generally close to 1 mÅ and 0.1°, respectively. As expected, both the uncertainties on the semi-experimental parameters and the errors of their PCS/Bonds counterparts are larger than those of the semirigid 2-furonitrile molecule. However, the PCS/Bonds errors on rotational constants are significantly reduced with respect to their rDSD/3F12^–^ and CCSD(T)/ccpVTZ counterparts.

### Cyclic and Bicyclic Compounds

**Figure 4:**
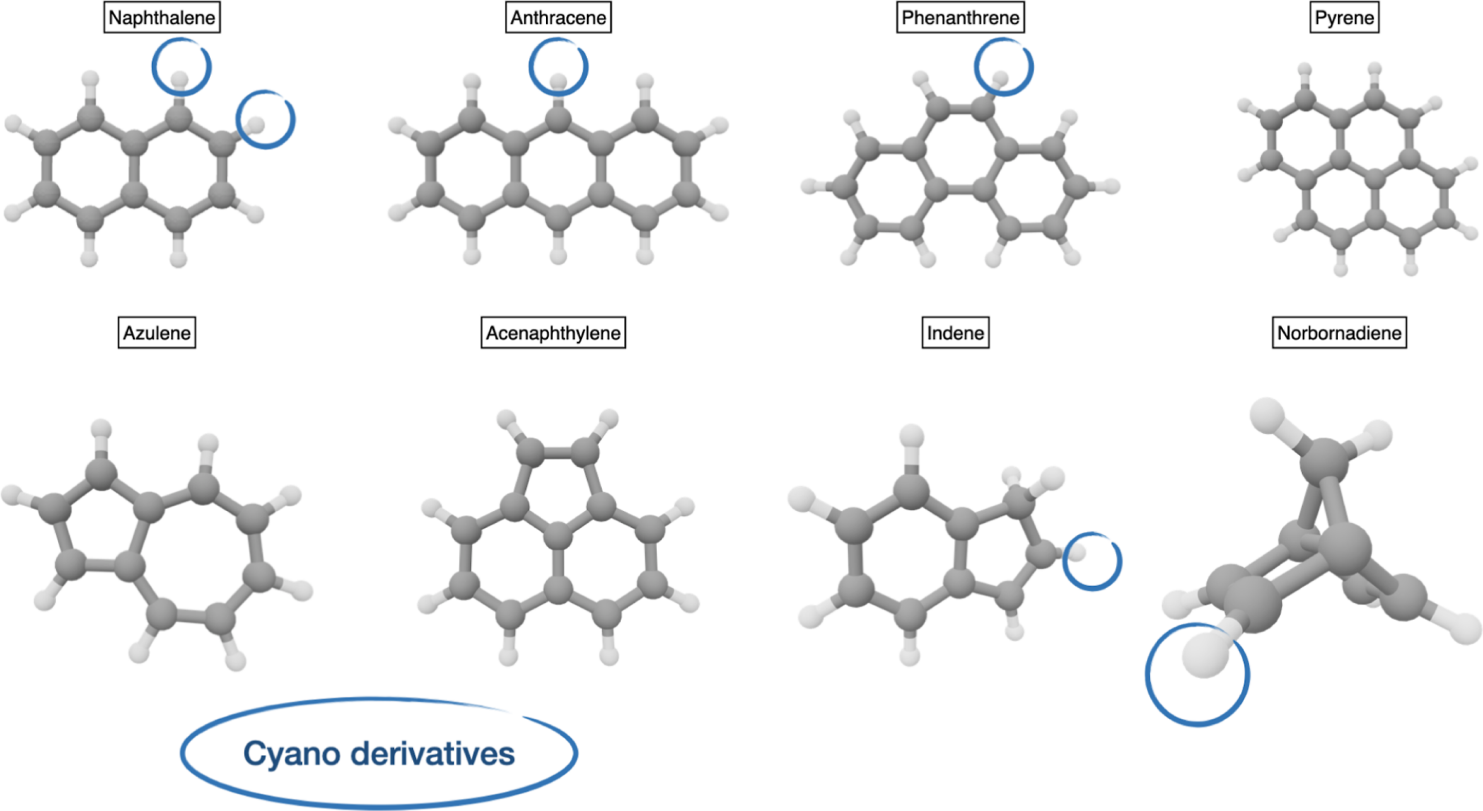
Structures of the PAHs studied in this work together with some CN-substituted species.

It is well recognized that PAHs and their nitrogen-containing PANH counterparts are widespread throughout the universe.^26^ Unfortunately, PAHs are either nonpolar or weakly polar (i.e., have a dipole moment lower than 0.5 Debyes), this preventing not only their radio-astronomical detection but also their laboratory characterization by rotational spectroscopy.^24,27,63^ In any case, ground-state rotational constants are available for several PAHs, and the corresponding experimental results are compared with their computed counterparts in Table 4, whereas the molecular structures are shown in Figure 4.

**Table 4:** Computed and Experimental Rotational Constants (in MHz) of Polycyclic Aromatic Molecules

molecule	param.	*B*_eq_ (rDSD)	Δ*B*_CVB_^a^	Δ*B*_VB_	Δ*B*_vib_	tot.	exp.
naphthalene	B_*a*_	3133.9	11.6(13.0)	–4.2	–22.7	3118.6	3119.4
	B_*b*_	1237.8	4.7(5.0)	–2.5	–7.7	1232.9	1233.0
	B_*c*_	887.3	3.4(3.7)	–1.3	–5.6	883.8	883.9
anthracene	B_*a*_	2155.3	8.0(8.5)	–3.0	–16.0	2144.3	2146.2
	B_*b*_	454.2	1.7(1.7)	–0.6	–2.7	452.6	452.5
	B_*c*_	375.1	1.4(1.7)	–0.5	–2.2	373.8	374.0
phenanthrene	B_*a*_	1622.0	6.0(6.5)	–2.3	–11.0	**1614.7**	1606.7
	B_*b*_	554.5	2.1(2.2)	–0.7	–3.6	**552.3**	547.7
	B_*c*_	413.2	1.6(1.7)	–0.5	–2.5	**411.8**	409.6
pyrene	B_*a*_	1015.3	3.9(4.2)	–1.4	–6.7	1011.1	1010.1
	B_*b*_	558.6	2.1(2.2)	–0.9	–3.4	556.4	556.5
	B_*c*_	360.4	1.4(1.3)	–0.5	–2.2	359.1	358.9
azulene	B_*a*_	2858.0	10.6(11.3)	–3.6	–20.1	2844.9	2842.0
	B_*b*_	1259.8	4.7(4.9)	–1.7	–7.7	1255.1	1254.8
	B_*c*_	874.4	3.3(3.4)	–1.2	–5.4	871.1	870.7
acenaphthylene	B_*a*_	1516.1	5.7	–1.0	–9.7	1511.1	1511.8
	B_*b*_	1224.8	4.6	–1.0	–7.7	1220.7	1220.6
	B_*c*_	677.5	2.5	–0.5	–4.2	675.3	675.5
indene	B_*a*_	3792.5	13.1	–2.1	–27.9	3775.6	3775.0
	B_*b*_	1584.7	6.0	–1.2	–9.5	1580.0	1580.9
	B_*c*_	1125.4	4.4	–1.0	–7.0	1121.8	1122.2
norbornadiene	B_*a*_	4305.7	16.6	–3.0	–40.6	4278.7	4273.6
	B_*b*_	3628.6	15.2	–1.5	–32.0	3610.3	3610.3
	B_*c*_	3199.4	11.5	–1.0	–24.0	3185.9	3186.4
MUE %^b^		0.410				0.042	
MAX %^b^		0.751				0.119	

a In parenthesis is the ΔCV2 contribution.

b Not including phenanthrene.

More recently, bicyclic compounds have been searched in the interstellar medium (ISM),^64^ and the prototypical norbornadiene molecule is included in the same table. Noted is that while naphthalene, anthracene, phenanthrene, and pyrene only involve six-membered aromatic rings, the situation is different for azulene, indene, and acenaphthylene. This aspect is not marginal since the very effective Lego brick approach^18^ mentioned in the Introduction cannot be applied to the last three molecules (and to norbornadiene) due to the lack of accurate structures for suitable fragments. The results collected in Table 4 show that the accuracy of the PCS/Bonds model is the same as that obtained for the validation set of molecules.

This finding is indeed remarkable since the dimensions of the considered PAHs are outside the range of application of state-of-the-art QC methods. The only outlier is phenanthrene, for which the relative error increases to 0.4%. Since there is no reason why the PCS/bonds and Lego brick^18^ approaches should perform well for anthracene and pyrene (and also for the CN derivative of phenanthrene, vide infra) and not for phenanthrene, we can only hypothesize that the experimental rotational constants are affected by some problem.^65^

**Figure 5:**
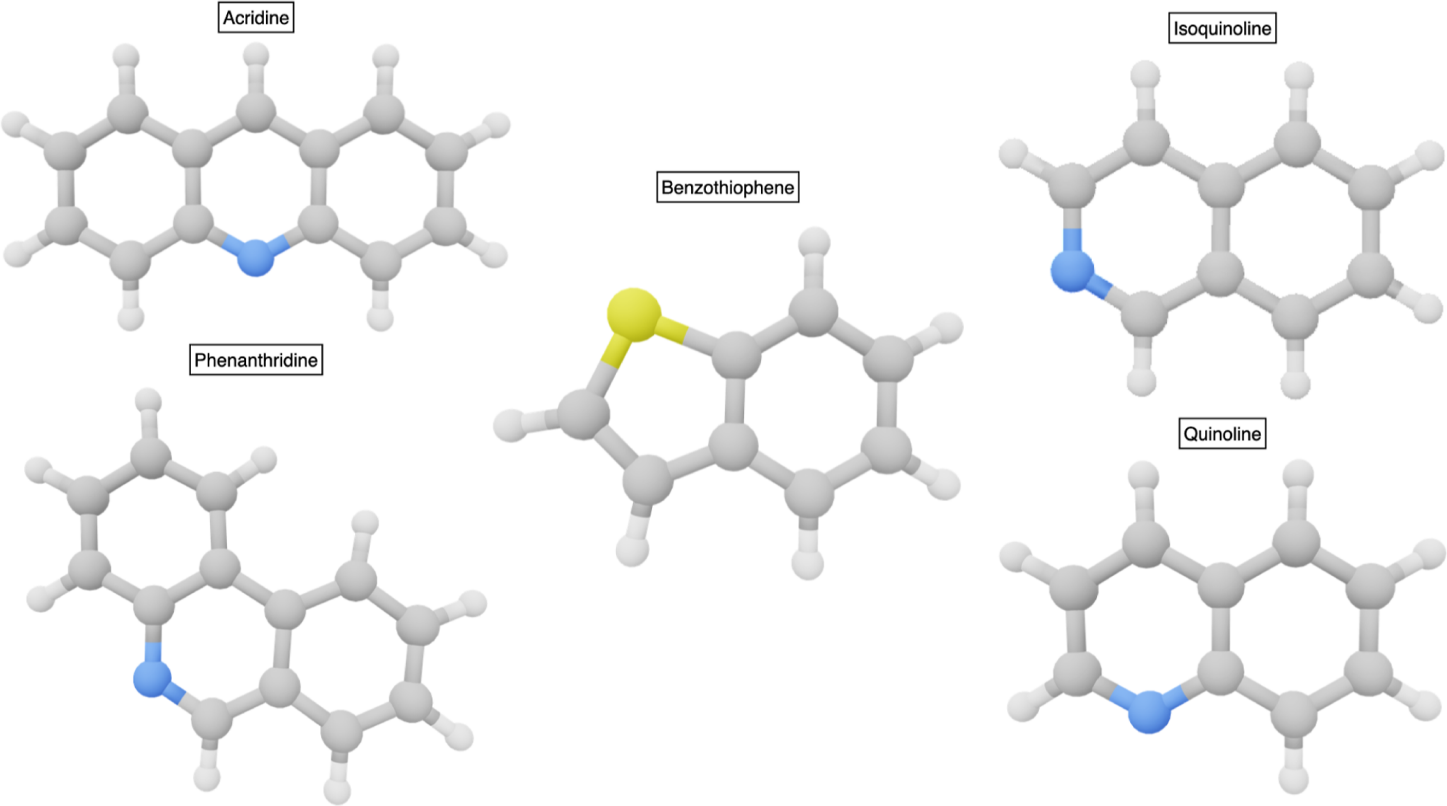
Heteroaromatic polycyclic molecules.

The issues related to the vanishing (or very small) dipole moments of most PAHs can be overcome by resorting to the cyano-substituted derivatives (CNPAHs), whose large permanent dipole moments produce intense rotational spectra. The abundance of the CN radical in the ISM^66^ and its extreme reactivity^27,63^ suggests that CNPAHs are good proxies of PAHs and can be used to guess the presence of the corresponding PAHs in the ISM. In the same vein, PANHs and other heterocyclic compounds have sufficiently large dipole moments and have been detected in several regions of the ISM. Furthermore, CN-substituted heteroaromatic and bridged bicyclic compounds have been recently searched^64^ in order to have an independent guess of the abundance ratio between CNPAHs and their parent PAHs. The structures of a panel of molecules belonging to all of these classes are shown in Figures 4 and 5, whereas the corresponding rotational constants are collected in Table 5.

**Table 5:** Computed and Experimental Rotational Constants (in MHz) of Polycyclic Heteroaromatic Molecules

molecule	param.	*B*_eq_ (rDSD)	Δ*B*_CVB_	Δ*B*_VB_	Δ*B*_vib_	tot.	exp.
quinoline	B_*a*_	3160.8	11.7	–3.9	–22.4	3146.2	3145.4
	B_*b*_	1276.3	4.8	–1.3	–7.9	1271.9	1271.6
	B_*c*_	909.2	3.4	–1.0	–5.7	905.9	905.7
isoquinoline	B_*a*_	3214.1	11.9	–3.6	–23.5	3198.9	3199.0
	B_*b*_	1242.8	4.7	–1.5	–7.5	1238.5	1237.9
	B_*c*_	896.3	3.3	–1.0	–5.6	893.0	892.8
acridine	B_*a*_	2163.3	8.0	–1.3	–16.0	2154.0	2154.4
	B_*b*_	468.0	1.8	–0.3	–2.7	466.8	466.6
	B_*c*_	384.8	1.5	–0.3	–2.2	383.8	383.6
phenanthridine	B_*a*_	1649.3	6.2	–1.1	–11.0	1643.4	1642.5
	B_*b*_	559.3	2.1	–0.4	–3.5	557.5	557.7
	B_*c*_	417.7	1.6	–0.3	–2.5	416.5	416.5
benzothiophene	B_*a*_	3165.6	12.6	–2.0	–21.2	3155.4	3153.8
	B_*b*_	1313.1	5.6	–0.9	–7.3	1310.5	1309.7
	B_*c*_	928.1	3.9	–0.6	–5.4	926.0	925.5
benzonitrile	B_*a*_	5681.4	21.9	–5.3	–40.4	5657.6	5655.3
	B_*b*_	1548.0	6.0	–1.0	–6.0	1547.0	1546.9
	B_*c*_	1216.6	4.6	–0.8	–5.8	1214.6	1214.4
3-cyano pyridine	B_*a*_	5852.3	21.7	–3.9	–39.5	5830.6	5823.1
	B_*b*_	1572.2	6.0	–0.8	–6.1	1571.3	1571.3
	B_*c*_	1239.3	4.7	–0.7	–5.8	1237.5	1237.2
1-cyano-naphthalene	B_*a*_	1483.8	5.8	–2.1	–8.2	1479.3	1478.9
	B_*b*_	959.4	3.5	–1.0	–5.3	956.6	956.8
	B_*c*_	582.7	2.1	–0.6	–3.2	581.0	581.0
2-cyano-naphthalene	B_*a*_	2717.6	10.2	–2.7	–17.8	2707.3	2707.0
	B_*b*_	607.2	2.3	–0.4	–3.1	606.0	606.1
	B_*c*_	496.3	1.9	–0.4	–2.6	495.2	495.3
9-cyano-phenanthrene	B_*a*_	848.5	3.2	–0.6	–4.9	846.2	846.1
	B_*b*_	487.6	1.9	–0.4	–2.8	486.3	486.4
	B_*c*_	309.7	1.2	–0.2	–1.7	309.0	308.9
9-cyano-anthracene	B_*a*_	987.3	3.6	–0.5	–5.1	985.3	985.8
	B_*b*_	452.8	1.7	–0.4	–2.8	451.3	451.2
	B_*c*_	310.4	1.2	–0.3	–1.7	309.6	309.6
2-cyano-indene	B_*a*_	3773.0	13.8	–2.8	–28.3	3755.7	3754.5
	B_*b*_	676.6	2.6	–0.4	–3.0	675.8	676.0
	B_*c*_	575.8	2.2	–0.4	–2.7	574.9	575.0
cyano-norbornadiene	B_*a*_	3854.3	14.3	–1.1	–32.0	3835.5	3831.7
	B_*b*_	1318.3	4.9	–0.4	–6.6	1316.2	1316.2
	B_*c*_	1243.2	4.7	–0.5	–6.0	1241.4	1241.8
MUE %		0.292				0.028	
MAX %		0.590				0.129	

**Figure 6:**
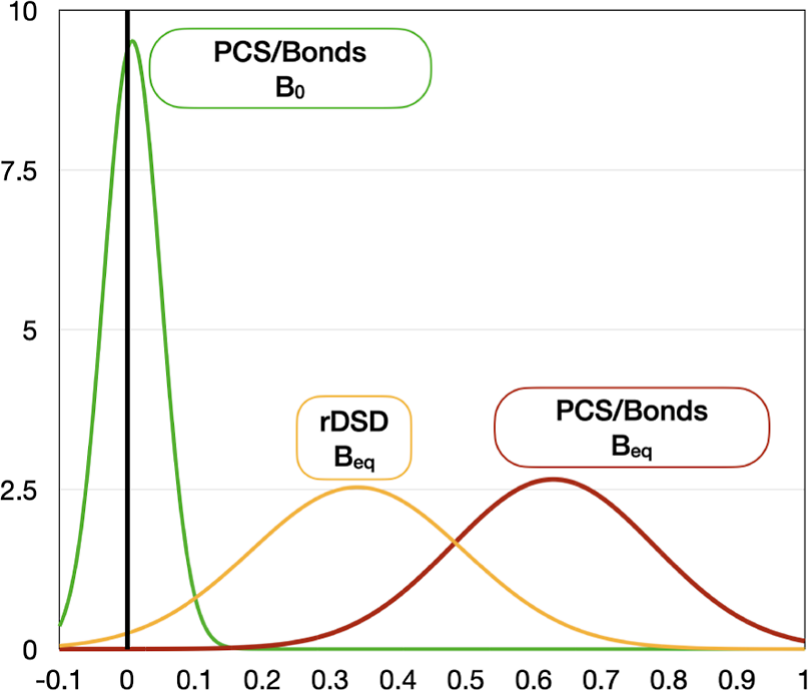
Error statistics for hydrocarbons (Table 4) and compounds containing heteroatoms (Table 5).

In general terms, the performances of the PCS/Bonds model are again comparable to those found for the validation set, and this can be better appreciated from the Gaussian distributions shown in Figure 6. These results confirm that the PCS/Bonds approach and the companion Web site permit the unbiased computation of accurate equilibrium geometries and rotational constants for semirigid molecules containing a few dozen atoms. Above these dimensions, the computation of vibrational corrections becomes the bottleneck of the whole procedure, and more effective approaches are under development in this connection.^22^ However, as already mentioned, the most critical issue is related to the flexibility of most molecules of biological interest. While the conformational search of stable structures can profit from multilevel QC methods possibly driven by Machine Learning algorithms,^67^ the underlying problem of large-amplitude motions limits the accuracy of any perturbative approach to vibrational corrections. Here, new ideas and implementations are surely needed.

## Conclusions

The main target of the present study was the computation of accurate geometrical parameters and rotational constants in the framework of a general strategy that aims to accurately characterize structural and spectroscopic properties of medium- to large-sized molecules. The main outcome is that accurate geometrical parameters can be computed by the PCS/Bonds model without any additional cost starting from geometries optimized by the revDSD-PBEP86-D3(BJ) functional in conjunction with the 3F12^–^ basis set. Then, the vibrational corrections needed for a proper comparison with experimental ground-state rotational constants can be effectively obtained from B3LYP-D3(BJ)/6-31+G* semidiagonal cubic force fields. In summary, the way seems to be paved toward the systematic study of molecules of astrochemical and/or prebiotic interest in the gas phase at a reasonable cost by an accurate black-box procedure.
